# Protective effects of a SIRT1 inhibitor on primordial follicle activation and growth induced by cyclophosphamide: insights from a bovine in vitro folliculogenesis system

**DOI:** 10.1007/s10815-022-02437-9

**Published:** 2022-03-05

**Authors:** Giovanna Di Emidio, Carla Tatone, Vincenza Barbato, Vincenzo Genovese, Martina Placidi, Riccardo Talevi, Roberto Gualtieri, on behalf of Italian Society of Embryology, Reproduction, Research (SIERR)

**Affiliations:** 1 Department of Life, Health and Environmental Sciences, University of L’Aquila, Building Delta 6, Via G. Petrini 6, 67100 L’Aquila, Italy; 2 Dipartimento di Biologia, Università di Napoli “Federico II”, Complesso Universitario di Monte S Angelo, Via Cinthia, 80126 Naples, Italy

**Keywords:** EX-527, SIRT1, HuR, In vitro folliculogenesis, Fertoprotective agents, Cyclophosphamide

## Abstract

**Purpose:**

Although oncological advances have improved survival rates of female cancer patients, they often suffer a reduced fertility due to treatment side effects. In the present study, we evaluated the potential fertoprotective effects of the specific inhibitor of SIRT1, EX-527, on the gonadotoxic action exerted by cyclophosphamide (CPM) on loss of primordial follicles (PFs).

**Methods:**

The effects of the CPM metabolite phosphoramide mustard (PM) on follicle activation, growth and viability and the protective action of EX-527 against PM effects were evaluated on bovine ovarian cortical strips in vitro cultured for 1 or 6 days. To understand whether PFs exposed to PM plus EX-527 were able to activate and grow to the secondary stage after suspension of the treatment, strips cultured for 3 days in PM plus EX-527 for 3 days were transferred to plain medium until day 6. Follicle growth and health were evaluated through histology and viability assay at a confocal microscope. In order to investigate the molecular pathways underlying the ovarian response to PM in the presence of EX-527, we analysed the protein level of SIRT1, HuR, PARP1 and SOD2 after 1 day of in vitro culture.

**Results:**

We found that (1) PM, the main CPM active metabolite, promotes PF activation; (2) the ovarian stress response induced by PM includes a SIRT1-dependent pathway; and (3) EX-527 reduces PF activation and growth induced by PM.

**Conclusion:**

SIRT1 can represent a candidate molecule to be targeted to protect ovarian follicles from alkylating agents and EX-527 could represent a potential fertoprotective agent for cancer patients.

## Introduction

Although oncological advances have improved survival rates of female cancer patients, after completing therapies many patients experience a reduced quality of life due to treatment side effects such as reduced fertility [1, 2]. In addition to their effects on ovarian follicle growth, chemo- and radiotherapies can damage the follicle reserve, the non-renewable stockpile of primordial follicles (PFs), with subsequent premature ovarian failure or insufficiency. Current options for fertility preservation in oncological female patients are based on assisted reproductive technologies (i.e. oocyte-embryo and ovarian tissue cryopreservation) [3]. Nevertheless, considering their limited applicability in relation to age and urgency of therapies, searching for fertoprotective medications is a pressing challenge in oncology practice [4]. To address this point, deep understanding of the mechanistic events that could be targeted to provide repair or protection from the gonadotoxic insult is needed.

Alkylating agents are the most toxic to the ovaries, and cyclophosphamide (CPM), which is routinely used against solid and haematological cancers, is associated with the highest rate of ovarian toxicity [5]. CPM can be considered a pro-drug since it requires hepatic biotransformation by cytochrome P-450 enzymes, to generate volatile metabolites and phosphoramide mustard (PM) [6–8]. In addition to targeting rapidly proliferating cells, CPM is capable of damaging quiescent cells [9].

Although CPM differently impacts on ovarian oocytes and somatic cells depending on animal age and follicle stage [10, 11], the mechanisms by which the quiescent PFs are affected remain to be established. Two main hypotheses have been proposed. They involve accelerated follicle activation due to direct targeting of the mechanisms regulating follicle dormancy (burnout) [12] including the lack of suppressive factors such as anti-Müllerian hormone (AMH) as a consequence of developing follicle degeneration [13]. The second hypothesis is based on the CPM-related induction of a damage response leading to apoptosis in human and oocytes from PFs and growing follicles following CPM exposure [14–16]. Relevant research in the animal model has highlighted the activation of molecular stress responses to DNA damage (DDR) and oxidative stress (OSR) induced by CPM [6, 8, 10, 17–22].

Di Emidio et al. [21] have provided strong evidence that an imbalance of redox potential is a main factor underlying CPM-induced ovarian damage and proposed the hypothesis that sirtuin 1 (SIRT1) plays a critical role in coordinating the ovarian stress response to CPM. SIRT1 is the founding and most well-studied member of the class III histone deacetylase family of enzymes that are dependent on nicotinamide adenine dinucleotide (NAD^+^) for their activity [23]. The control of SIRT1 expression involves all the major points of regulation, including transcriptional and post-translational mechanisms and microRNAs [24]. SIRT1 is a stress‐responsive enzyme that orchestrates cellular adaptations by altering the acetylome. As a result of the variety of nuclear and cytoplasmic proteins modulated by lysine acetylation, SIRT1 has been shown to be a master regulator of cellular activities such as gene expression, energetic metabolism, differentiation, apoptosis, DNA repair, senescence and oxidative stress response. The role of SIRT1 in oxidative stress and redox signaling has been well-established in numerous tissues and organs including the ovary [25–28]. It has been observed that mild oxidative stress conditions induce the expression of SIRT1, thus affecting SIRT1 targets that are involved in the adaptive response. On the contrary, exposure to harsh oxidative stress results in increased proteasomal degradation and/or enzyme inactivation of SIRT1 and reduced survival [26, 29]. In mouse oocytes exposed to mild oxidative stress, SIRT1 expression increases and protects them by activating enzymatic antioxidant defences [30]. Interestingly, a role for SIRT1 as a regulator of the PF activation process has recently been demonstrated [31]. Therefore, SIRT1, being a regulator of both follicle dormancy and ovarian stress response to CPM, is a good candidate as a sentinel molecule directly involved in the damage of ovarian reserve by alkylating agents.

By virtue of these considerations, the principal aim of the present study is to evaluate the potential beneficial effects of EX-527, a specific inhibitor of SIRT1, on the action exerted by CPM on dormancy, activation and growth of PFs, along with the underlying molecular mechanisms. To this aim, a well-established in vitro culture of bovine ovarian cortical strips in gas-permeable dishes has been adopted as an in vitro folliculogenesis system [32].

## Materials and methods

### Chemicals and consumables

Lumox gas-permeable 50-mm culture dishes (PD) were purchased from Sarstedt (NuÈmbrecht, Germany). Leibovitz’s L-15, α-MEM GlutaMax, Insulin-Transferrin-Selenium (ITS) 100 × and Live/Dead Fixable Far Red Stain were purchased from Invitrogen (Milan, Italy). l-Ascorbic acid, l-glutamine, bovine serum albumin (BSA), penicillin–streptomycin (PEN/STREP), amphotericin B, Hoechst 33,342, fructose, α-thioglycerol, eosin-Y, radio-immunoprecipitation assay (RIPA) buffer, polyvinylidene difluoride (PVDF) membrane, Tris-buffered and saline Tween 20 were purchased from Sigma Aldrich (Milan, Italy). Paraffin wax and Mayer’s haematoxylin were from Carlo Erba (Milan, Italy). Bicinchoninic acid (BCA) protein assay kit was from Pierce (Rockford, IL, USA). Anti-SIRT1 antibody (Ab7343), anti-superoxide dismutase 2 (SOD2) antibody (Ab86087) and horseradish peroxidase (HRP) conjugated anti-mouse secondary antibody (Ab6728) were purchased from Abcam (Cambridge, UK). Mouse monoclonal anti-human antigen R (HuR) antibody (SC-71290) and anti-poly [ADP-ribose] polymerase 1 (PARP-1) antibody (sc-74479) were purchased from Santa Cruz Biotechnology Inc. (Dallas, TX, USA). Anti-glyceraldehyde-3-phosphate dehydrogenase (GAPDH) antibody (TA802519) was purchased from OriGene Technologies Inc. (Rockville, MD, USA). HRP-conjugated anti-rabbit antibody (BA1054) was purchased from Boster Biological Technology Co., Ltd (Pleasanton, CA, USA). ECL kit was purchased from Thermo Scientific (Waltham, MA, USA).

### Collection and preparation of ovarian tissue

Bovine ovaries were collected at the time of slaughter from individuals aged 8–24 months (Slaughterhouse Straccione, San Marcellino, Caserta, Italy; CEE accreditation number 1403/M) and transported within 2 h to the laboratory in Leibovitz’s L-15, 2 mM glutamine, 3 mg/mL BSA, 1% Pen-Strep and 1 μg/mL amphotericin B (handling medium) at 4 °C. Cortical slices (~ 0.5 mm thick) were manually dissected from bovine ovaries in handling medium at room temperature (RT), avoiding regions with visible antral follicles. Cortical slices were homogeneously minced into 1 mm × 1 mm × 0.5 mm strips using a tissue chopper (McIlwain, Mickle Laboratory Engineering Company, Ltd, Surrey, UK), pooled and mixed by gentle agitation in a 10-cm Petri dish and washed twice in fresh handling medium, and 10 cortical strips were randomly distributed into 5 mL of medium for culture in PD. Fresh strips from each sample were processed for histology and viability assessment as controls.

### Culture of ovarian tissue in gas-permeable dishes

In each experiment, strips from the same ovary were cultured in PD in α-MEM, 3 mM glutamine, 0.1% BSA, 1% Pen-Strep, 1% ITS (10 μg/mL Insulin, 5.5 μg/mL Transferrin, 6.7 ng/mL Selenium), 1 μg/mL amphotericin B, 50 μg/mL ascorbic acid at 37 °C, 5% CO_2_ and 95% humidity in air for 1, 3 and 6 days. These time points were selected on the basis of previous studies [32] showing a considerable activation of primordial follicles and progression to the secondary stage after 3 and 6 days of culture respectively. Culture for 1 day was carried out to detect early activation of signalling pathways under different experimental conditions in accordance with previous research [21]. Half medium was changed every 48 h. At the end of culture, 5 strips from each dish were treated for histology and 5 strips for viability assessments. Parallel samples were treated for SDS page and western blotting as detailed below.

### Experimental design

Experiment 1 (*n* = 3) was aimed to evaluate the effects of the active cyclophosphamide (CPM) metabolite PM (10 µM [8, 33]) on follicle activation, growth and viability and the possible protective action of the specific SIRT1 inhibitor 6-chloro-2,3,4,9-tetrahydro-1H-carbazole-1-carboxamide (EX-527) on PM effects. To this aim, ovarian strips were cultured for 6 days in (1) medium alone (6D); (2) PM 10 µM (PM 6D); (3) EX-527 10 µM (EX 6D); and (4) PM 10 µM plus EX-527 10 µM (PM + EX 6D).

Experiment 2 (*n* = 3) was aimed to understand whether PFs exposed for 3 days to PM plus EX-527 were able to activate and grow during culture in medium alone for 3 additional days. Ovarian strips were cultured in (1) medium alone for 3 days (3D) or (2) 6 days (6D); (3) PM 10 µM plus EX-527 10 µM for 3 days (PM + EX 3D); and (4) PM 10 µM plus EX-527 10 µM 3 days followed by culture in medium alone for 3 days (PM + EX 3D + M 3D).

Experiment 3 (*n* = 3) was aimed to evaluate the effects of exposure to PM, EX-527, PM plus EX-527 or medium alone on expression of SIRT1, HuR, PARP1 and SOD2. To this end, ovarian strips were cultured as in experiment 1 for 1 day and then processed for protein extraction, SDS page and western blotting. Parallel samples were processed for histology and viability.

### Histology

Cortical strips were fixed in Bouin’s, dehydrated in increasing ethanol concentrations and embedded in paraffin and 5 μm serial sections were stained with haematoxylin and eosin. Grading and staging of follicles were performed on serial sections by two blinded expert observers and only follicles in which the germinal vesicle was present in the section were counted. Follicle quality was scored as previously described (see [32, 34] for further details and representative images of follicle classes) and follicle stages were classified according to van Wezel and Rodgers [35].

### Viability assessment

Strips were incubated under shaking in 1 μg/mL Live/Dead Fixable Far Red Stain and 10 μg/mL Hoechst 33,342 in Dulbecco’s PBS for 3 h at 4 °C, fixed in 4% paraformaldehyde in Dulbecco’s PBS for 2 h at RT, washed in fresh buffer and incubated at 4 °C overnight in 10 μg/mL Hoechst 33,342 in buffer [32, 36]. Strips were then optically cleared as previously reported [37]. Strips were mounted in 115% fructose on a glass slide with 3 spacer coverslips (0.17 mm) placed on each side and covered with a coverslip. Analysis was carried out with a Leica TCS SP5 confocal scanning laser microscope (Leica Microsystems, Wetzlar, Germany) using a 405 nm diode laser for visualization of the nuclear label (Hoechst 33,342) and a 633 nm helium neon laser for the live/dead probe. Each strip was analysed using the z-position control and imaged to a depth of 300 μm from the tissue surface using a 63 × glycerol immersion objective.

### Western blot analysis

Ovarian strips were homogenised in RIPA buffer by repeated freeze/thaw cycles in liquid nitrogen followed by sonication for 30 s on ice. After centrifugation (14,000 rpm for 90 min at 4 °C), the supernatants were collected and stored at − 80 °C until processed for protein analysis. Protein concentration was determined by BCA protein assay kit. Thirty micrograms of proteins was resolved by 10% sodium dodecyl-sulphate polyacrylamide gel electrophoresis (SDS-PAGE) electrophoresis, transferred to a PVDF membrane and blocked with 5% BSA in Tris-buffered saline containing 0.05% Tween 20 (TBS-T) for 1 h at room temperature. Then, membranes were incubated with polyclonal rabbit anti-SIRT1 antibody (1:700), anti-SOD2 antibody (1:1000), mouse monoclonal anti-HuR (1:250), anti-PARP-1 (1:300) or anti-GAPDH (1:750) overnight at 4 °C, followed by incubation with HRP-conjugated anti-rabbit (1:3000) or anti-mouse secondary antibody (1:2000) for 1 h at room temperature. After washing, the immunoreactive bands were stained by ECL kit and immediately detected by Uvitec Cambridge system (Alliance Series, Cambridge, UK). The images obtained by Uvitec were analysed by ImageJ 1.44p software to obtain values of band intensity. Data obtained were placed in a spreadsheet. The intensity of bands of the proteins of interest was normalised for GAPDH and values were given as relative units (RU). The experiment was performed in triplicate.

### Statistical analysis

Histological and viability data are presented as cumulative percentages. Statistical analysis was performed by Fisher’s exact test for pairwise comparisons when overall significance was detected. Western blotting data are presented as mean ± SEM. Statistical analysis was assessed by *t* test or one-way ANOVA followed by the Tukey HSD test for multiple comparison. Analyses were performed using the SigmaStat software (Jandel Scientific Corporation). *p* values < 0.05 were considered statistically significant.

## Results

### Experiment 1

**Fig. 1:**
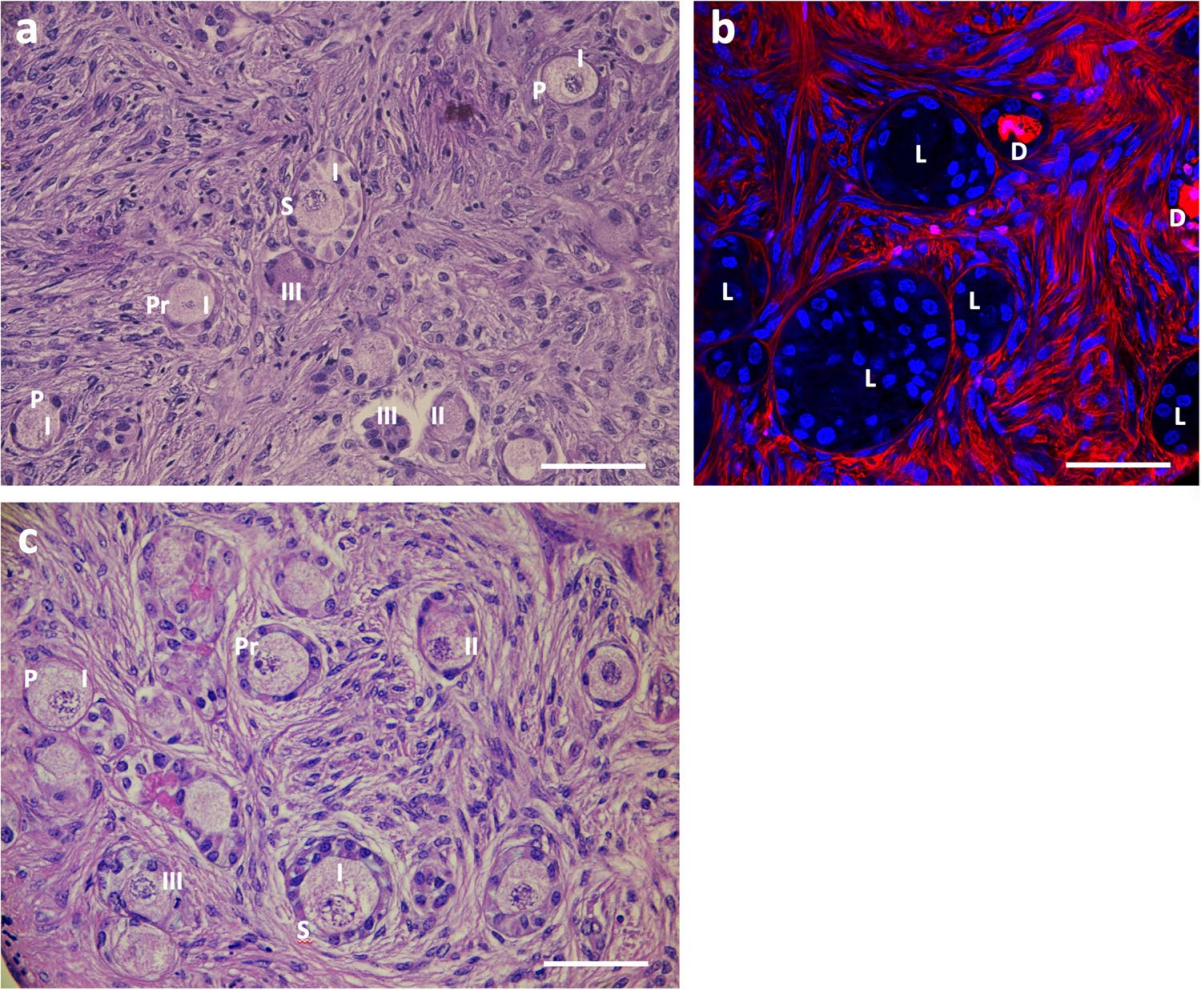
Representative micrographs of follicle stage and quality (**a**, **c**), and viability (**b**) in strips cultured for 6 days in medium (**a**, **b**) or in medium plus phosphoramide mustard (**c**). **a**, **c** Hematoxylin/eosin histological section. P, primordial follicle; Pr, primary follicle; S, secondary follicle; I, grade I follicle; II, grade II follicle; III, grade III follicle. **b** Confocal image of strips labelled with Live/Dead Far Red (red) and Hoechst 33,342 (blue). L, live follicle; D, dead follicle. Bar, 50 μm

**Fig. 2:**
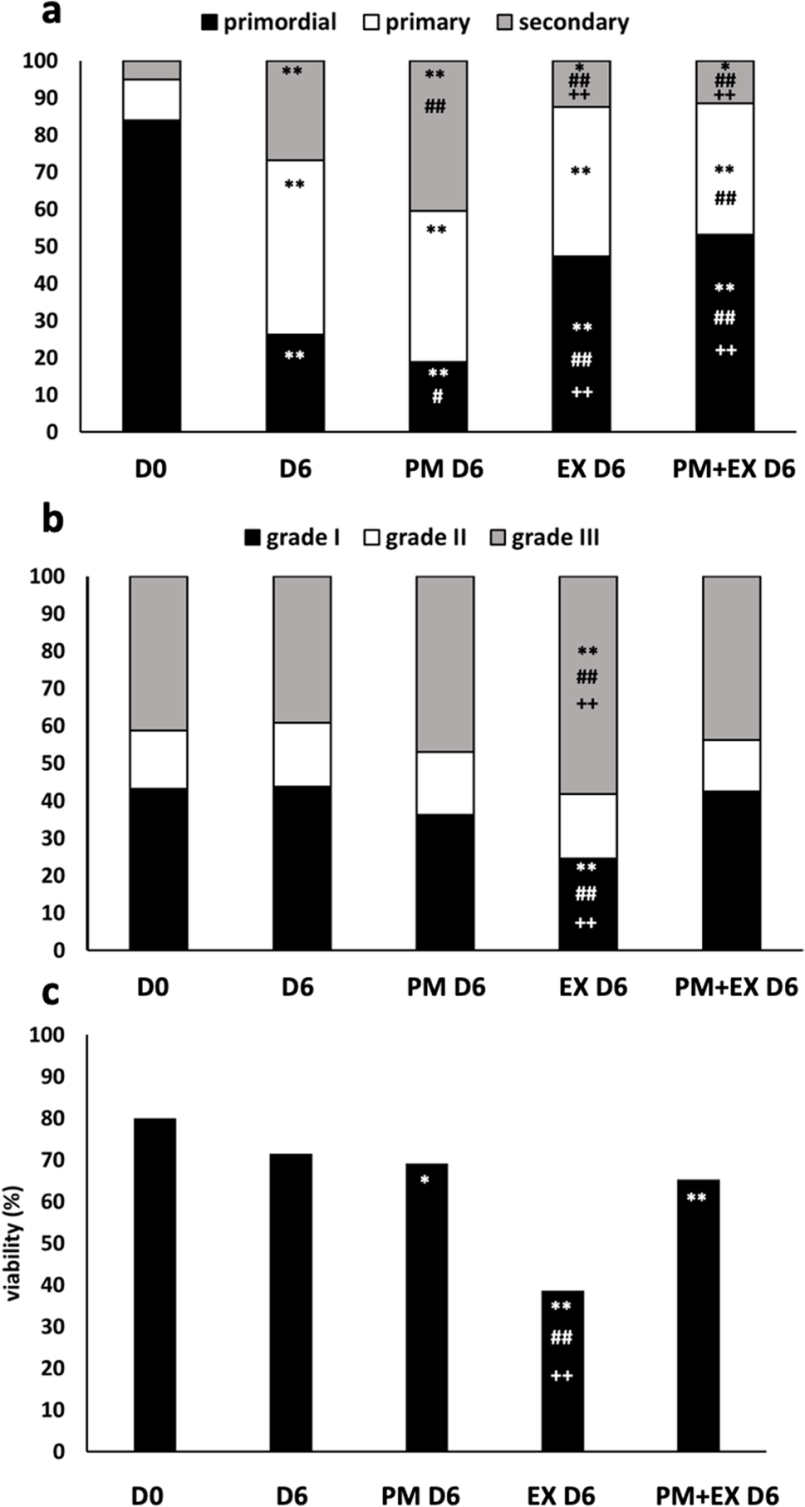
Histological staging (**a**), grading (**b**) and viability (**c**) of bovine follicles in fresh ovarian tissue (D0) and ovarian cortical strips cultured for 6 days in medium (D6), phosphoramide mustard (PM D6), EX-527 (EX D6) or phosphoramide mustard plus EX-527 (PM + EX D6). Statistical analysis on cumulative percentages by Fisher’s exact test for pairwise comparisons. *and **, *p* < 0.05 and *p* < 0.01 vs D0; # and ##, *p* < 0.05 and *p* < 0.01 vs D6; + and +  + , *p* < 0.05 and *p* < 0.01 vs PM D6

Experiment 1 (*n* = 3) was aimed to evaluate the effects of PM and EX-527 used alone or in combination on follicle activation, growth and health during bovine ovarian tissue in vitro culture. Overall, follicle staging (Figs. 1a, c and 2a) and grading (Figs. 1a, c and 2b) were assessed on 1397 follicles, and viability (Figs. 1b and 2c) on 1216 follicles. At day 0 (D0), most follicles were primordial (primordial, 84%; primary, 11%; secondary, 5%). Culture for 6 days (D6) decreased the proportion of PFs and increased that of primary and secondary stage follicles (primordial, 26%; primary, 47%; secondary, 27%) (Fig. 2a), as an evidence of activation of PFs and the progression to growth phase. Culture with PM for 6 days (PM D6; Fig. 1c) promoted a significantly higher follicle activation and growth to the secondary stage compared to culture for 6 days in medium alone (primordial, 18%, *p* < 0.05; secondary, 40%, *p* < 0.01). Culture with EX-527 for 6 days (EX D6) reduces PF activation and growth (primordial, 47%; secondary, 12%) compared to both control day 6 (*p* < 0.01) and PM D6 (*p* < 0.01).

Follicle quality (Fig. 2b) was not significantly affected by culture in medium alone, PM and PM + EX, except for EX that induced a highly significant decrease of grade I follicles and increase of grade III follicles compared to day 0, tissue cultured in PM + EX, or in medium alone (*p* < 0.01).

Follicle viability (Fig. 2c) in tissues cultured in PM and PM + EX did not differ from samples cultured in medium alone, though a slight but significant reduction was observed compared to day 0 (PM vs D0, *p* < 0.05; PM + EX vs D0, *p* < 0.01). In sharp contrast, a marked decrease of follicle viability was observed in tissues cultured in EX-527 compared to D0, D6, PM D6 and PM + EX D6 (*p* < 0.01). Overall, data indicates that PM enhances follicle activation and growth, and the combined treatment with EX-527 is able to prevent them without affecting follicle quality and viability. Although treatment with EX-527 alone is also able to prevent follicle activation and growth, it exerts a detrimental action on follicle quality and viability with respect to its use in conjunction with PM.

### Experiment 2

**Fig. 3:**
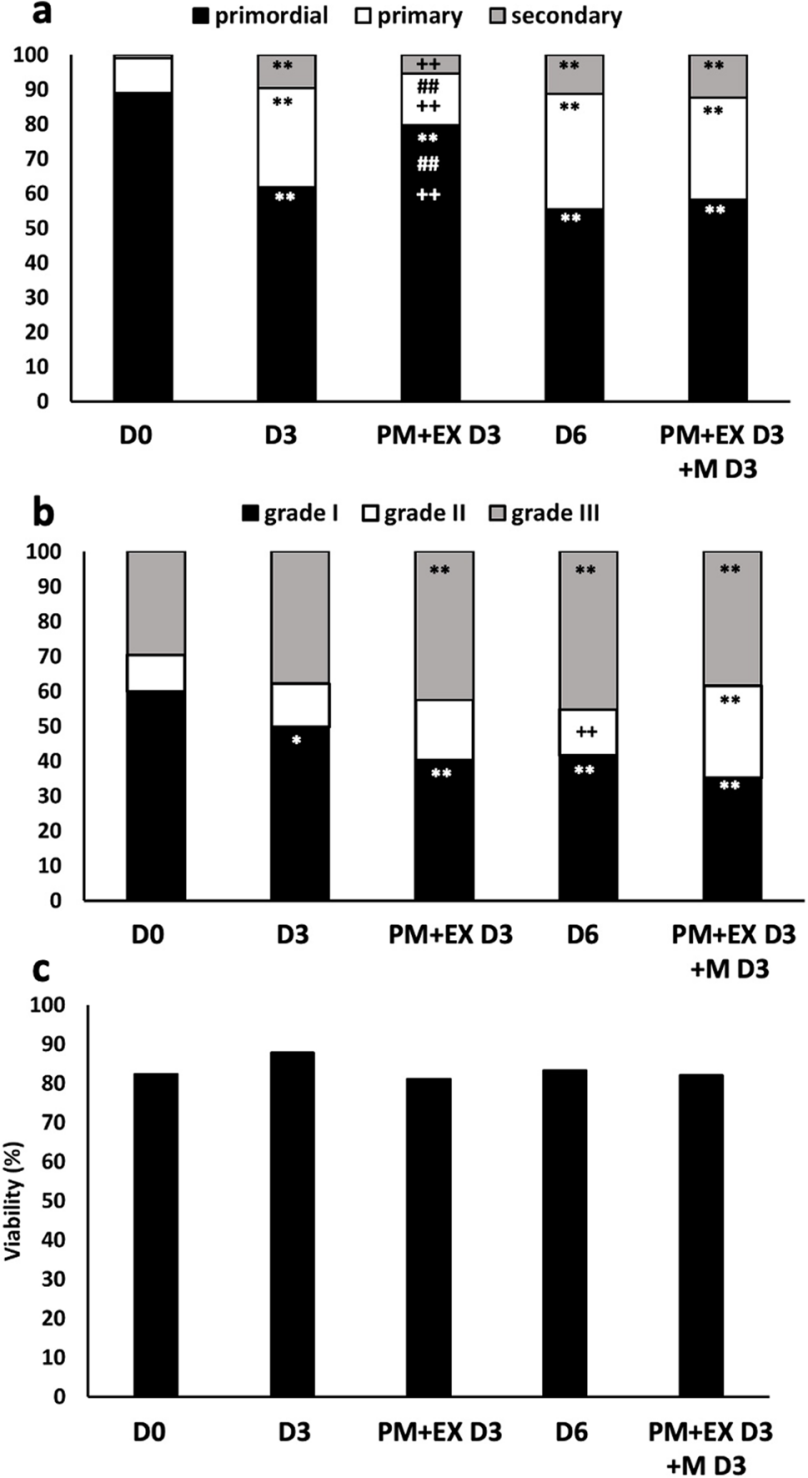
Histological staging (**a**), grading (**b**) and viability (**c**) of bovine follicles in fresh ovarian tissue (D0) and ovarian cortical strips cultured 3 (D3) or 6 days (D6) in medium, 3 days in phosphoramide mustard plus EX-527 (PM + EX D3) or 3 days in phosphoramide mustard plus EX-527 followed by culture in medium for further 3 days (PM + EX D3 + M D3). Statistical analysis on cumulative percentages by Fisher’s exact test for pairwise comparisons. *and **, *p* < 0.05 and *p* < 0.01 vs D0; # and ##, *p* < 0.05 and *p* < 0.01 vs D3; + and +  + , *p* < 0.05 and *p* < 0.01 PM + EX D3 + M D3

The experiment (*n* = 3) has been addressed to understand whether follicles whose activation and growth were prevented by combined treatment with PM and EX-527 were able to activate and grow to the secondary stage after suspension of the treatment. To this end, bovine ovarian tissue was cultured in medium for 3 (D3) or 6 days or under combined treatment with PM and EX-527 for 3 days (PM + EX D3), followed or less by culture in medium alone for further 3 days (PM + EX D3 + M D3). Overall, follicle staging (Fig. 3a) and grading (Fig. 3b) were assessed on 1485 follicles, and viability (Fig. 3c) on 1032 follicles. Culture in medium for 3 days led to a significant follicle activation and growth (D0 vs D3: primordial, 89 vs 61%; primary, 10 vs 28%; secondary, 1 vs 9%) that was prevented by culture in PM + EX-527 for 3 days (PM + EX D3 vs D3: primordial, 79 vs 61%; primary, 15 vs 28%; secondary, 5 vs 9%). Culture in medium alone for 3 days of tissue exposed to the combined treatment for the previous 3 days allowed follicle activation and growth (PM + EX D3 + M D3 vs PM + EX D3: primordial, 58 vs 79%; primary, 29 vs 15%; secondary, 12 vs 5%) to values similar to those recorded in tissue cultured in medium alone for 6 days. No differences in follicle quality were detected in samples cultured for 3 days with PM plus EX-527 compared with parallel samples cultured for 3 days in medium alone. Samples cultured with PM plus EX-527 for the first 3 days and then in medium alone for 3 more days (PM + EX D3 + M D3) had a significant increase of medium quality follicles (grade II) and a corresponding decrease (not significant) of grade I follicles compared to parallel samples cultured for 6 days in medium alone. Viability did not differ amongst samples.

### Experiment 3

Experiment 3 (*n* = 3) was aimed to investigate the molecular pathways underlying the ovarian response to PM observed after a 6-day in vitro culture in the presence or absence of the SIRT1 inhibitor. To this end, we analysed the protein level of SIRT1, HuR, PARP1 and SOD2 after a 1-day in vitro culture of bovine ovarian strips. Parallel samples were processed to evaluate follicle staging and health after culture for 1 day (D1).

**Fig. 4:**
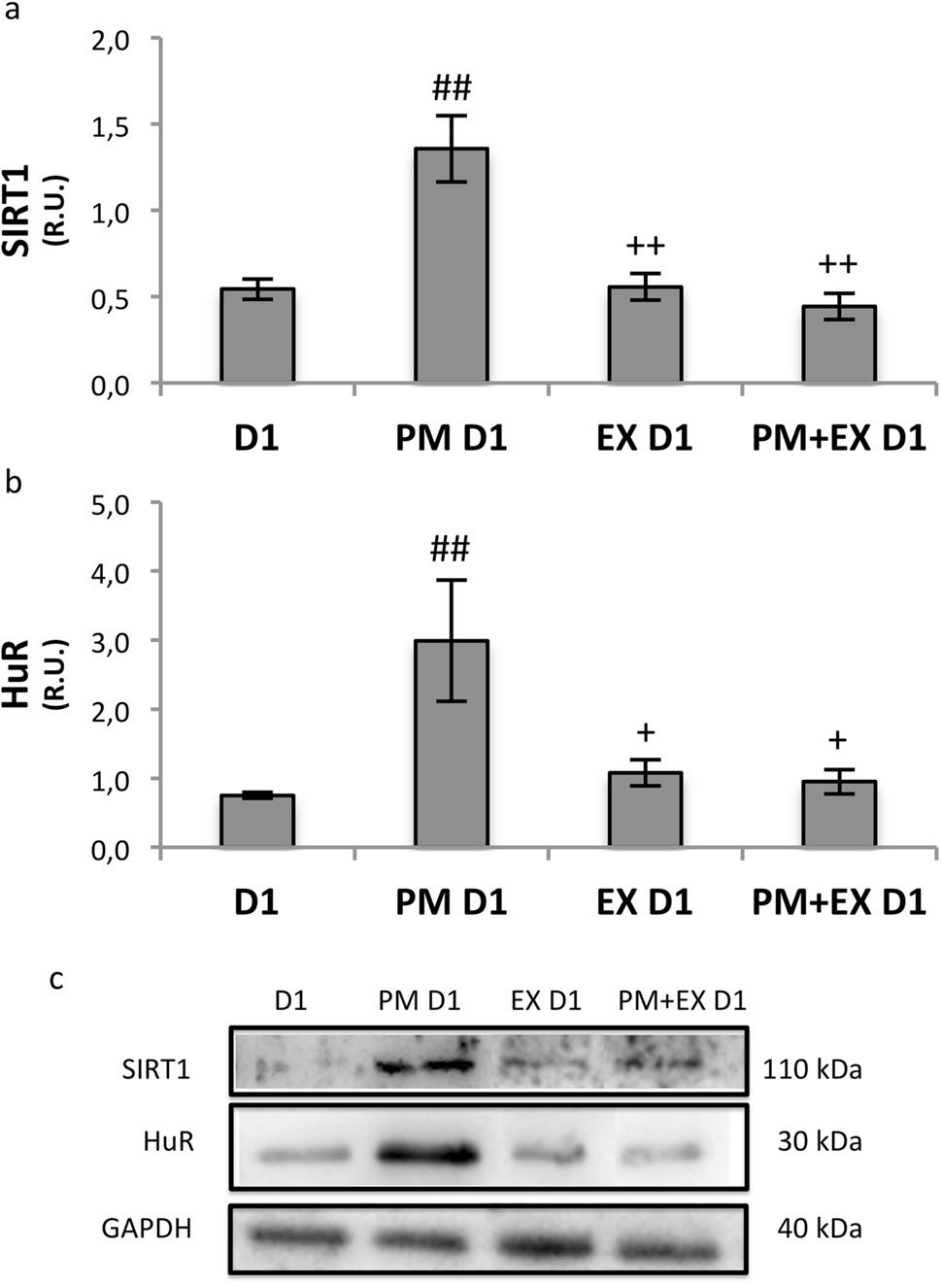
Western blot analysis of SIRT1 (**a**) and HuR (**b**) and representative images of immunoreactive bands (**c**). Data are presented as means ± SEM of densitometric analysis of immunoreactive bands normalised to internal reference protein (GAPDH). Statistical analysis by one-way ANOVA, followed by the Tukey HSD test. #*p* < 0.05 vs D1, ##*p* < 0.01 vs D1; + *p* < 0.05 vs PM D1, +  + *p* < 0.05 vs PM D1

Western blot analysis showed that PM treatment significantly increased SIRT1 protein after 24 h. Moreover, our results disclosed that SIRT1 remained at basal levels when EX-527 was present revealing that the PM-induced SIRT1 increase was prevented by the presence of the SIRT1 inhibitor (Fig. 4a). The SIRT1 protein level in the presence of EX-527 alone was similar to control D1. Regarding HuR, present results displayed that PM treatment induced an increase in HuR protein level (Fig. 4b). By contrast, HuR expression in the presence of EX-527 was maintained at basal levels independently from the presence of PM.

**Fig. 5:**
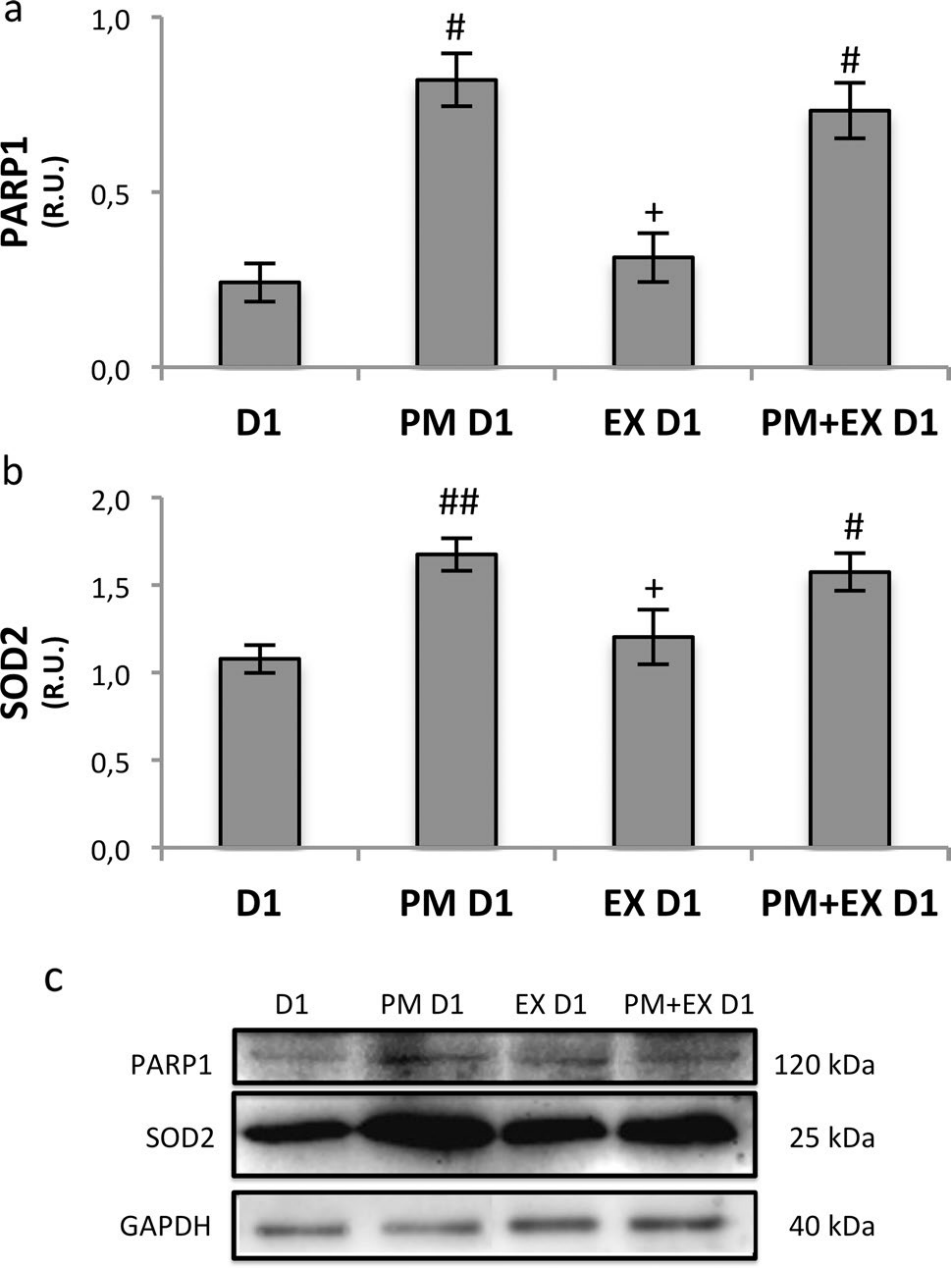
Western blot analysis of PARP1 (**a**) and SOD2 (**b**) and representative images of immunoreactive bands (**c**). Data are presented as means ± SEM of densitometric analysis of immunoreactive bands normalised to internal reference protein (GAPDH). Statistical analysis by one-way ANOVA, followed by the Tukey HSD test. #*p* < 0.05 vs D1, ##*p* < 0.01 vs D1; + *p* < 0.05 vs PM D1, +  + *p* < 0.05 vs PM D1

To test whether ovarian adaptive response to PM involves DDR and/or OSR signalling, we analysed the protein expression level of PARP1 and SOD2, as main components of DDR and OSR, respectively (Fig. 5). As shown in the figures, both PARP1 and SOD2 significantly increased as early as 24 h (D1) upon the PM insult indicating that both DDR and OSR are activated. Moreover, the results show that PARP1 and SOD2 protein levels after PM exposure were not affected by the SIRT1 inhibitor. The exposure to EX-527 alone did not alter PARP1 and SOD2 expression levels which were similar to control (Fig. 5).

**Fig. 6:**
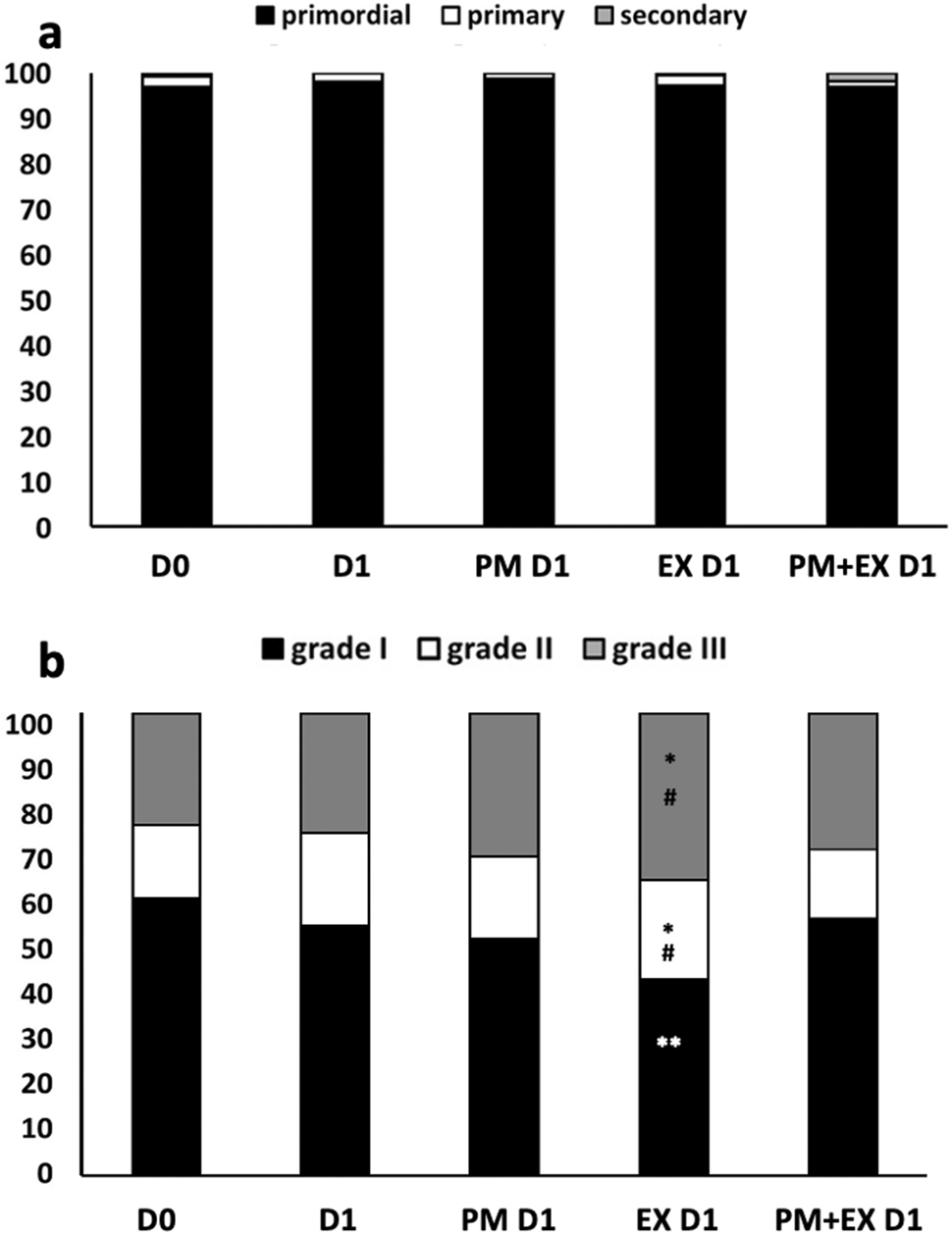
Histological staging (**a**) and grading (**b**) of bovine follicles in fresh ovarian tissue (D0) and ovarian cortical strips cultured for 1 day in medium (D1), phosphoramide mustard (PM D1), EX-527 (EX D1) or phosphoramide mustard plus EX-527 (PM + EX D1). Statistical analysis on cumulative percentages by Fisher’s exact test for pairwise comparisons. *and **, *p* < 0.05 and *p* < 0.01 vs D0; # and ##, *p* < 0.05 and *p* < 0.01 vs D1

Overall, follicle staging (Fig. 6a) and grading (Fig. 6b) were assessed on 1540 follicles, and viability on 1307 follicles. At D0, most follicles were primordial (primordial, 97; primary, 2; secondary, 1%). As expected, culture for 1 day (D1) did not promote the activation of PFs and the progression to the primary and secondary stage under all conditions tested (Fig. 6a).

Follicle quality (Fig. 6b) was not significantly affected by culture in medium alone, PM and PM + EX, except for EX that induced a significant decrease of grade I follicles compared to D0 and a significant increase of grade II and III follicles compared to both D0 and PM D1 (*p* < 0.01). Follicle viability (not shown) exceeded 90% in all samples.

## Discussion

Anticancer therapy is known to be associated with increased risk of premature ovarian failure (POF) and infertility. As an early effect, gonadotoxic treatment induces amenorrhea and the loss of follicles, and the long-term is represented by the permanent loss of the ovarian reserve as recently reviewed [38]. Therefore, it urges to set up fertoprotective strategies suitable for patients of all ages and avoid the use of invasive hormonal and surgical procedures [4]. So far, animal models have strongly contributed to define molecular mechanisms underlying chemotherapy cytotoxicity and the search for effective pharmacological options to prevent follicle loss at the time of treatment [4].

Herein, the bovine has been adopted as a model for human folliculogenesis as in both species one dominant follicle is selected from a cohort of 5–30 follicles, all other recruited follicles become atretic, the time of development from primordial to the ovulatory follicle stage is similar, and the number of early growing follicles slowly declines with increasing age [35, 39, 40]. Therefore, in vitro culture of bovine ovarian cortical strips in gas-permeable dishes has been adopted as an in vitro folliculogenesis system to investigate whether SIRT1, a NAD^+^-dependent deacetylase with numerous regulatory roles in the ovary [26], can be proposed as a molecule to be targeted to protect follicles from alkylating agents. The main findings of the present study are the following: (1) PM, the main CPM active metabolite, promotes PF activation; (2) the ovarian stress response induced by PM includes a SIRT1-dependent pathway; (3) the SIRT1 inhibitor EX-527 reduces PF activation and growth induced by PM.

### The main CPM active metabolite, PM, accelerates bovine in vitro PF activation and growth

As previously demonstrated, the culture system used in this study efficiently supports PF activation and enhances the rate of secondary follicles developed after in vitro culture of bovine ovarian cortical strips by means of optimization of oxygen availability through in vitro culture in gas-permeable dishes [32]. Under these conditions, here we observed a reduction of PFs in the control group and an increase in growing follicles as early as 3 days in in vitro culture, as an evidence of in vitro PF activation which further increases at 6 days from the beginning of the culture. This phenomenon could be ascribed to Hippo signalling disruption in response to the tissue fragmentation as reported in the mouse and human [41, 42]. Exposure to PM for 6 days was associated with decreased proportion of PFs and higher percentage of growing follicles when compared with untreated follicles. This supports the hypothesis that PM induces follicle ‘burnout’ as described for the first time in murine in vivo model [12] and further observed in human ovarian tissue in vitro [33]. Based on the analysis of follicle quality and viability here reported, follicles exposed to PM for 1 day or 6 days are viable and maintain a normal morphology suggesting that under our experimental conditions PM does not exert any evident detrimental effect on bovine follicles. Our observations are in agreement with Lande et al. [33] who showed that follicle activation in response to PM was not associated with DNA fragmentation in human ovarian strips cultured in vitro. By contrast, previous findings in rodent models indicate that PM negatively affects follicle viability in in vitro cultured neonatal ovaries [6, 8] with specific effects on pre-theca and pre-granulosa cells which, however, were shown to be resistant to DNA damage–induced apoptosis [11]. Consistent follicle damage with no evidence of follicle burnout was found in a human ovarian xenograft model at least in the first 12 h after CPM administration [16]. Overall, these observations provide evidence that the experimental model, exposure time and species affect follicle response to CPM and its metabolites.

### The ovarian stress response induced by PM includes a SIRT1-dependent pathway

The hypothesis that SIRT1 is involved in the ovarian response to CPM arises from the finding that that CPM administration induces early upregulation of ovarian SIRT1 protein in the mouse. We have previously demonstrated that the ovary activates an adaptive response based on upregulation of SIRT1 protein that reaches its peak at 24 h [21]. Therefore, here we analysed the protein level of SIRT1 after a 1-day in vitro culture of bovine ovarian strips exposed to PM in the presence or absence of EX-527. Our results show that PM treatment significantly increased SIRT1 protein. Moreover, the PM-induced SIRT1 increase is prevented by the presence of the SIRT1 inhibitor, suggesting that SIRT1 activity is required for increasing SIRT1 expression under stress conditions [26]. In many tissues, SIRT1 abundance can be regulated by modulating the mRNA stability by the RNA-binding protein HuR (Hu antigen R). Indeed, HuR stabilises SIRT1 transcripts and promotes their polyribosome engagement for active translation [43]. Consistently, PM treatment of ovarian cortical strips induces an increase in HuR protein expression level in accordance with our previous observations in granulosa cells and murine ovaries [21]. We have also observed that HuR increase induced by PM is prevented by SIRT1 inhibition. This suggests that HuR is strictly involved in the ovarian adaptive response to PM by participating in a positive feedback loop regulating SIRT1 expression. Nevertheless, other roles for HuR cannot be excluded since it can stabilize a wide number of transcripts bearing AU-rich elements whose turnover is critical for cell fate [44, 45].

### SIRT1 inhibitor EX-527 protects against in vitro PF activation induced by PM

EX-527 is a specific SIRT1 inhibitor showing 100-fold selectivity for SIRT1 over SIRT2/3, along with metabolic stability and membrane penetrability [46]. We report here that the presence of the SIRT1 inhibitor EX-527 results in an increased proportion of PFs when compared with untreated follicles following a 6-day in vitro culture. Moreover, this effect was observed either in the presence or absence of PM indicating that SIRT1 inhibition is a general mechanism disrupting ovarian follicle quiescence in our culture system independently of the stimulus. However, in contrast to EX-527-exposed follicles challenged with PM, the quality and viability of follicles exposed to the SIRT1 inhibitor alone were consistently reduced. This is in accordance with the observations that, in somatic cells, treatment with EX-527 is beneficial under conditions stimulating SIRT1 expression (for a review, see [47]), as it occurs in ovarian strips exposed to PM. By contrast, consistent with the key role of SIRT1 as a regulator of cell fate, prolonged SIRT1 inhibition under physiological conditions hampers follicle quality and viability. Accordingly, previous work demonstrated that continuous exposure of mouse oocytes to EX-527 interferes with in vitro maturation and promotes ROS production [30]. On the other hand, EX-527 in the presence of PM does not negatively affect follicle competence. The absence of detrimental effects in the follicles whose activation and growth were prevented by combined treatment with PM and EX-527 for 6 days is confirmed by the observation that they were able to activate and grow to the secondary stage after suspension of the treatment without evident effects on quality and viability.

Based on the considerations above, our findings are consistent with recent research demonstrating the role of SIRT1 as a regulator of the PF activation process in the mouse model [31]. Indeed, either SIRT1 activation or upregulation results in increased activation of PFs, whereas SIRT1 knockdown in oocytes or PF cells significantly suppresses this process [31]. Moreover, Cinco et al. [48] observed increased nuclear expression of SIRT1 in mouse oocytes during the transition from the primordial to the primary follicle in concomitance with decrease in the NADH/NAD^+^ ratio, a condition favouring SIRT1 activation. Overall, SIRT1, being a regulator of both follicle dormancy and ovarian stress response to CPM, is a good candidate as a sentinel molecule directly involved in the loss of ovarian reserve induced by alkylating agents.

### Ovarian adaptive response to PM involves components of DDR and OSR signalling

To test whether ovarian adaptive response to PM involves DDR and/or OSR signalling, we analysed the protein expression level of PARP1 and SOD2, as main components of DDR and OSR, respectively. PARP1 regulates primary repair mechanisms to resolve DNA lesions and plays a critical role in the maintenance of chromosome stability at key stages of meiosis in the female germ line [49–52]. PARP1 is strictly linked to intracellular NAD^+^ level, and its hyper-activation may deplete intracellular NAD^+^ and ATP levels [53]. In rat neonatal ovaries, PM-induced activation of DDR genes and proteins including PARP1 was observed as early as 12 h after in vitro exposure [19, 20]. SOD2 is a mitochondrial antioxidant enzyme involved in the first defence against free radicals. It catalyses the conversion of superoxide anions to hydrogen peroxide, a major type of ROS, which is further turned to water by CAT (catalase) or GSH-Px (glutathione peroxidase), so playing a key role in maintaining the cellular redox balance and ROS scavenging in the ovary [54]. Here we have reported that both PARP1 and SOD2 significantly increased as early as 24 h upon the PM insult, indicating that both DDR and OSR are activated. We can speculate that the maintenance of high PARP1 and SOD2 levels under SIRT1 inhibition may favour the survival of a higher percentage of PF in comparison with PM alone. This hypothesis is in accordance with the recent finding that PARP1 activity is essential for PF preservation [55].

## Conclusions

Here we proposed for the first time the hypothesis that SIRT1 can be a candidate as a molecule to be targeted to protect ovarian follicles from alkylating agents. This conclusion is based on the findings that the ovarian stress response induced by PM includes a SIRT1-dependent pathway and that the SIRT1 inhibitor EX-527 reduces PF activation and growth induced by PM. Nevertheless, further studies are needed to better characterise the beneficial effects of EX-527 against the molecular damage by CPM. Moreover, that this inhibitor does not counteract the anticancer effect exerted by this alkylating agent remains to be demonstrated. EX-527 therapeutic potential has been evaluated in animal models for several pathologies, including cancer. EX-527 safety was demonstrated in phase I clinical trials and it is currently under investigation as a therapeutic agent in women with unexplained infertility (Trial NCT04184323). Taken together, these data support the potential use of EX-527 as a fertoprotective agent for cancer patients.

## Data Availability

The data presented in this study are available on request from the corresponding author.
